# Academic Stress and Physical Activity in Adolescents

**DOI:** 10.1155/2020/4696592

**Published:** 2020-02-24

**Authors:** Karel Frömel, Michal Šafář, Lukáš Jakubec, Dorota Groffik, Radim Žatka

**Affiliations:** ^1^Faculty of Physical Culture, Palacký University Olomouc, třída Míru, Olomouc 771 11, Czech Republic; ^2^Institute of Sport Sciences, The Jerzy Kukuczka Academy of Physical Education in Katowice, Mikołowska 72a, Katowice 40-065, Poland

## Abstract

The issue of work-related mental health needs to be addressed at the school level. The aim of this study was to explore the associations between academic stress (AS) of adolescent boys and girls and their physical activity (PA) during recesses and after school and to propose measures to promote the adoption of lifelong healthy working habits. Adolescents from 16 schools in the Czech Republic and 6 schools in Poland participated in the study (187 boys and 339 girls). Monitoring of PA and cognitive stress was conducted during one school day. We used ActiTrainer accelerometers to monitor PA and physical inactivity. Data on time of PA and self-reported AS in school lessons were collected using recording sheets. We split the participants into two groups: those without a self-reported stressor and those who indicated one or more stressors. Differences in overall PA during recesses, as well as after-school PA, between boys with and without AS were not statistically significant for any PA characteristics. We observed similar results for girls. Repeated measures ANOVA confirmed that differences in PA (steps/hour) during recesses following particular lessons between participants with and without AS were not statistically significant in boys or girls (*F*_(4,1612)_ = 1.83, *p*=0.121 , *η*_*p*_^2^=0.005). It is noteworthy that girls with AS were statistically significantly more likely to meet the 6000 steps after school time recommendation (39%) than girls without AS (18%; *p* < 0.001). The study did not confirm the assumption that adolescents reporting AS have less PA during recesses or even after school than adolescents without AS. However, the overall low PA of adolescents during recesses and after school highlights the need to compensate for AS by adequate PA. This is especially true for adolescents with recurrent AS in several consecutive lessons.

## 1. Introduction

In the context of insufficient and declining levels of physical activity among adolescents [1, 2] and inadequate physical fitness [3, 4], the high cognitive and emotional load of adolescents in schools is negatively associated with their mental health (MH) [5]. Although some studies have not confirmed the association between physical activity (PA) decline and changes in MH in adolescents, this does not undermine the importance of studying these associations [6]. The prevalence of mental disorders in 12- to 17-year-olds in the USA has been increasing [7, 8], as is the case in Europe [9] and other countries [10]. In addition, while the prevalence of mental disorders is generally high, there exists significant inequality in MH among young people [11].

Mental disorders occur more frequently in younger than older individuals [12]. Thus, such disorders will often occur in lower levels of school. Mental health and behavioral problems in childhood predict similar problems in adolescence [13]. Half of all mental health problems in adulthood are manifested in adolescence or before [9]. In schools in the United Kingdom [14], Germany [15], and other countries, the deterioration of pupils' MH is a current challenge, consistent with The Lancet Commission's call to urgently address this undesirable trend [16]. It is possible that the education system, school environment, and education system itself have a deleterious effect on the mental health of pupils.

Academic stress (AS) is a serious issue [17]. We consider AS in line with Selye's [18] understanding of this term as an individual's nonspecific set of responses to internal or external stimuli intervening with the habitual academic process. AS is a topic of interest also because it needs to be considered in relation to future work-related stress. AS can be understood analogically to work-related stress. It is mostly acceptable to define work-related stress “as a negative psychological state with cognitive and emotional components with effects on the health of both individual employees and their organizations” ([19]: 12). Psychosocial, environmental, safety, organizational, and other aspects of work-related stress are of the same importance when addressing the issues of AS in the school environment.

The severity of the negative impact of AS is probably the most critical in Asian countries [20]. Despite this, Zhao et al. [21] note that reformation of the Chinese education system, which aimed to reduce academic stress, has not yet been effective. These researchers highlighted the importance of raising the level of education in both China and the United States; however, with respect to global economic competition, the issue also concerns sustaining a humanistic approach to learning. Yoo [22] expresses similar concerns about the psychosocial development of South Korean adolescents, on the basis of the finding that bonding social capital is a causal predictor of AS. At the same time, he indicated that it is necessary to consider that the effect of bonding social capital on AS may be either negative or positive.

Criticism of deteriorating MH in schools must be viewed in the context of state, school, and health policies, with cooperation between school authorities, school management, teachers, parents, representatives of municipalities, leisure-time educators, and other staff involved in education. Cross-national cooperation of schools across Europe is therefore very important, given the complexity of addressing MH issues in schools [23]. This is especially so because other factors interfere with the MH of children and youth. These factors not only relate to physical health, demographics, and environment but also concern socioeconomic or ethnic factors, individual personality traits, and other public-health aspects. Gender differences play an important role in adolescents' MH in school [24], particularly with respect to mental resilience. However, there is limited evidence regarding the longer-term effects of interventions that aim to increase mental resilience [25]. For these and other—particularly developmental—reasons, the issue of the children's and youth's MH is more complicated than physical health regarding diagnostics and treatment of symptoms, and more difficult in adulthood. At the same time, it is clear that the period of school attendance, and hence the role of the school, is critical in preventing MH issues in children and youth [26].

Evidence of the effectiveness of interventions into MH crises in schools is very limited [27]. Greater effectiveness is likely following the promotion of MH literacy, as shown by Millin et al. [28], who demonstrated the effectiveness of MH literacy among high school students. However, there is a lack of peer-reviewed literature explaining the importance of MH literacy for MH enhancement [29].

Little is known about the associations between the physical and mental conditions of the younger generation. However, their mutual dependence is clear. In particular, cardiorespiratory fitness can be an important indicator of health, including mental health, in children and youth [30]. PA is also effective in improving self-esteem and reducing depression, but it does not affect anxiety scores [31]. It has also been shown that vigorous PA, cardiorespiratory fitness, and BMI are associated with adolescents' mental well-being and quality of life [32]. Improved physical fitness in adolescence may be associated with fewer symptoms of depression [33] or may reduce their social physique anxiety [34]. Physical inactivity associated with stress might also be detrimental to adolescents' physical health, including increasing the risk of injuries [35]. Together with other risk and organizational factors, this amplifies cardiovascular risk in the workplace [36] as well as in schools.

School policy, curricula, and educational processes are unable to respond to changes in the lifestyle of children and youth which is confirmed by the requirements for reforms in education systems among European countries [37]. A school environment that meets the needs of children and youth should serve as the foundation of the future work environment. Therefore, significant competencies should include strategies to compensate mental load, such as PA coupled with mental relaxation.

Therefore, the aim of the current study was to explore the associations between academic stress of adolescent boys and girls and their physical activity during recesses and after school and to propose measures to promote the adoption of lifelong healthy working habits.

## 2. Methods

### 2.1. Participants

The research was carried out between 2015 and 2016 in sixteen schools in the Czech Republic and six schools in Poland. Due to the complexity of PA monitoring, schools were selected on the basis of extant long-term research collaboration. Overall, 187 boys (36 Polish boys) and 339 girls (51 Polish girls) met the criteria to participate in the study (Table 1). In this sample, we analyzed 1122 school lessons and recesses that met the criteria for PA monitoring (boys without AS, *n* = 244, and with AS, *n* = 95; girls without AS, *n* = 547, and with AS, *n* = 236). Given the similarities in the types of schools, the organization of the teaching process (the same duration of lessons and recesses), and the sampling of participants, we did not include the factor of the country in the analyses. For individual analysis of lessons (congruent in Czech and Polish schools) and recesses, we chose six participants who self-reported AS in four or more lessons. Those were the only participants who indicated AS in four or more lessons and showed a high heart rate (≥60% HRmax - minutes), while being physically inactive. The selected participants represent an extremely stressed group with the highest health risk.

### 2.2. Assessment of Physical Activity

For PA monitoring, we used the ActiTrainer™ accelerometer (Florida, USA; http://www.theactigraph.com/products/actitrainer), which measures PA (counts) and heart rate (HR). As part of the initial training, the participants registered in the web application (http://www.indares.com). During registration, the participants provided basic data about themselves, of which we used information on age, weight and height, country, day of monitoring in the week, size of the place of residence, ownership of a dog in their family, and participation in organized PA. The training covered exploration of the Indares features, how to wear the accelerometer, measurement of resting heart rate (HRrest), and supplementary recordings (course of the day, records of PA duration and type).

Immediately after waking in the morning, the participants measured their HRrest three times for 15 seconds and noted their beats per minute. The resulting HRrest was calculated as the mean of the three measured values and then controlled using the lowest daily HR value recorded by the ActiTrainer device. HR measurements were cross-checked using a Polar S610iTM heart rate monitor upon participants' arrival in school. The participants launched the actual PA monitoring the next morning (after morning hygiene) and wore the accelerometer throughout the day (except for bathing and swimming) until going to bed. This routine was the same in the next two days. We included the first day with complete and flawless PA and HR records in the analyses (Monday: 19 days; Tuesday: 61 days; Wednesday: 42 days; Thursday: 53 days; and Friday: 32 days).

During the day, the participants recorded the times of events before school, at school in accordance with the schedule of lessons and recesses, and after school. In the evening, after the end of the monitoring day, they wrote down the type and duration of all types of physical activity and inactivity (lasting at least 10 minutes) carried out during the day. These data were used only to verify the objective data obtained from the accelerometers.

To process the accelerometer data (15-second epoch), we used the custom software IntPA13 (https://upol.cz/fileadmin/userdata/FTK/Fakulta/Verejnost/Navod_IntPA13.pdf), which is only available in the Czech language. The program is able to assess the duration of PA and physical inactivity in specific school-day segments. PA intensity was determined using HR data, which was expressed in 10% intervals between 30% and100% of HRmax (for boys, HRmax = 220 - age and for girls, HRmax = 226 - age) and in MET units as positive integers. Intensity zones were divided into low (50–59.9% HRmax; <3 METs), moderate (60–84.9% HRmax; 3–5.9 METs), and vigorous PA (85–100% HRmax; ≥6 METs). In this study, we present results concerning moderate-to-vigorous PA (MVPA) intensity (60–100% HRmax, ≥3 METs). The accelerometer data were processed consistent with previously published methods [38–41].

Only the participants who provided a valid record of at least three lessons in school, at least 120 minutes of after-school time, and at least 480 monitoring minutes throughout the day were included in the analyses. The number of physical education lessons was similar in both groups of participants (25% without AS and 28% with AS). We excluded 89 participants who fail to meet these criteria.

After the research was complete, all participants received individual feedback on their PA and physical inactivity, caloric expenditure, HR, and step count. The feedback contained information on PA intensity in METs and HR zones, together with simple line graphs presenting daily caloric expenditure and HR [41]. School management received aggregate anonymized results of the entire study sample.

### 2.3. Record of Time Segments and Stressors in Lessons

After the end of each lesson, the participants provided a self-assessment regarding excessive (greater than habitual) AS experienced during the course of the lesson. The term AS was explained and defined to the participants as negative stress (distress) during an initial session with them. The major stressors in lessons were represented only by external negative factors (oral examination, written tests, time-demanding schoolwork, overall work overload, negative actions of a teacher or student, the inadequate difficulty of organization or cooperation, and other academic tasks exceeding the adaptive capacities of the participants). If it occurred, self-assessed negative stress was reported in recording sheets next to particular lessons (clearly defined types of lessons: mathematics, geography, foreign language, etc.) using the “S” mark. Participants also provided verbalized (written) explanations of AS, particularly the main causes of the self-reported individual AS. These stress records were then counter-checked with the heart rate records. Objectively measured heart rate was higher than the resting heart rate, with participants being physically inactive, in all lessons marked with “S.” According to the stress records, we split the participants into a group that did not record AS in any lesson (without AS) and a group with AS at least one lesson (with AS).

### 2.4. Statistical Analysis

Statistica version 13 (StatSoft, Prague, Czech Republic) and SPSS version 22 (IBM Corp., Armonk, NY) were used for statistical analyses. First, we computed basic descriptive statistics. Next, we used the Kruskal-Wallis test to analyze differences in aggregate PA of different groups of participants, repeated measures ANOVA to assess differences in PA on individual days, and contingency tables to assess differences in compliance with PA recommendations. Finally, binary logistic regression with the standard enter method (all independent variables are entered into the equation at the same time) was used to assess the odds of meeting after-school PA recommendations. The *η*^2^ and *η*_*p*_^2^ effect size coefficients were evaluated as follows: 0.01 ≤ *η*^2^ < 0.06 was considered a small effect size, 0.06 ≤ *η*^2^ < 0.14 was considered a medium effect size, and *η*^2^ ≥ 0.14 was considered a large effect size.

### 2.5. Ethics

The management of each school, all participants, and their parents received information about the objectives and course of the research, in respect of the fact that they provided written informed consent. These persons were informed of the provision of the individual feedback on the participants' individual results and that anonymity would be preserved in the aggregate group results and future publication of the overall research results. The Indares web application conforms with the requirements of the General Data Protection Regulation (EU 2016/679). The study was approved by the Institutional Research Ethics Committee of the Palacký University of Olomouc (decision no. 24/2012).

## 3. Results

### 3.1. Analysis of Differences in Boys' and Girls' PA between Those with and without AS

Differences in the overall PA between boys, as well as girls, with and without AS were not statistically significant for any of the PA characteristics (Table 2). Only differences in the overall PA during recesses were statistically significant, when we compared boys and girls with AS. Girls with AS reached 30.6 min·hour^−1^ of PA during recesses in contrast to boys who reached 37.0 min·hour^−1^.

Additionally, none of the differences in after-school PA among boys, as well as girls, with or without AS were statistically significant for any of the PA characteristics (Table 3). Differences in steps/hour and MVPA (≥3 METs) were statistically significant between boys and girls with AS.

Repeated measures ANOVA confirmed that the differences in PA (steps/hour) during recesses between the participants with and without AS were not statistically significant in boys or girls (*F*_(4,1612)_ = 1.83, *p*=0.121, *η*_*p*_^2^=0.005). Only differences between boys and girls reporting AS were statistically significant, with boys being more physically active (*F*_(4,1612)_ = 2.87, *p*=0.022, *η*_*p*_^2^=0.007). The most pronounced differences were found during the second recesses (usually 20 minutes long), in which boys without AS reached 546 ± 769 steps/hour (girls 367 ± 467 steps/hour), whereas boys who reported AS reached 540 steps/hour (girls 374 ± 420 steps/hour).

### 3.2. Meeting PA Recommendations in Boys and Girls with and without AS

**Figure 1:**
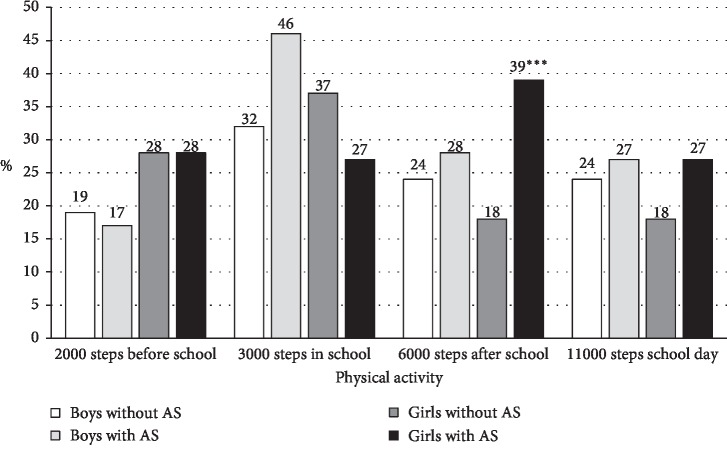
Proportions of boys (*n* = 187) and girls (*n* = 339) with and without academic stress (AS) who met the physical activity recommendations in school-day segments.

A significantly higher number of girls with AS met the after-school PA recommendation (39%) than girls without AS (18%; *p* < 0.001, Figure 1). In contrast, a larger proportion of girls without AS (37%) met the recommendation for PA in school (3000 steps/school time) than girls experiencing AS (27%), but the difference was not statistically significant despite reaching practical significance. Concerning the 11,000 steps/day recommendation, we did not observe statistically significant differences between the groups with and without AS.

The association between AS and compliance with the recommendation of 6000 steps after school time was also confirmed by the results of the binary logistic regression, in both girls and boys (Table 4). The odds of meeting the 6000 steps after school time recommendation in boys and girls with AS was not significantly influenced by age, BMI, participation in organized PA, country, day of the week, size of the place of residence, or dog ownership. Meeting of the 6000 steps/after-school time was primarily associated with boys' participation in organized PA (*p*=0.025).

### 3.3. Individual Evaluation of Participants Reporting AS in Four or More Lessons (Group with the Highest Health Risk)

**Figure 2:**
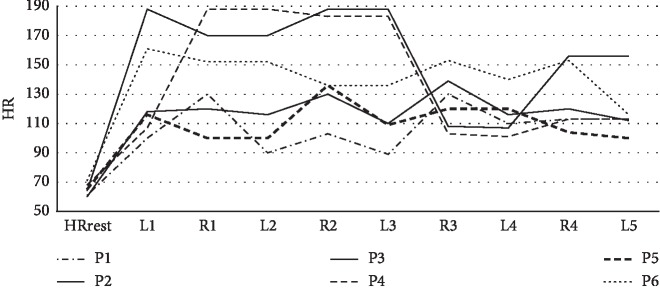
Maximum heart rate achieved in lessons (L1–L5) and subsequent recesses (R1–R4) in six participants (P1–P6) who reported academic stress in four or more lessons. HR: heart rate; HRrest: resting heart rate.

**Figure 3:**
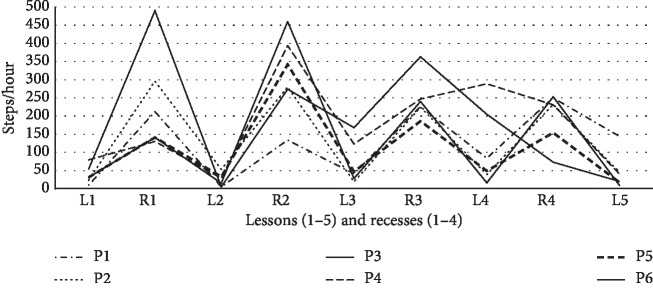
Mean number of steps per hour in lessons (L1–L5) and subsequent recesses (R1–R4) in six participants (P1–P6) who reported academic stress in four or more lessons. L: lesson; R: recess.

The HRmax achieved in the lessons and subsequent recesses highlights the high level of mental stress in six participants who reported AS in four or more lessons (Figure 2); while being physically inactive, we detected high HR that was confirmed by self-reported AS. HR recorded in recesses showed apparent gradual fading of mental stress after the lesson; however, PA (steps/hour) of these most “stressed” adolescents was similar to that of the other participants (Figure 3). Overall, PA and MVPA accounted for 55.4% and 12.3% of the total recess time, respectively, in the most “stressed” participants (54.7% and 7.5% in the entire sample).

### 3.4. Self-Assessment of AS in Lessons

Out of 526 days of PA monitoring, participants reported self-assessed AS beyond that habitually experienced during the course of lessons on 207 days. The main causes of AS in lessons were written tests and exams (61.4%), oral examinations (16.9%), and demanding lesson content (10.6%). A small proportion (10.1%) of participants also reported fear, especially of the teacher. We did not find any statistically significant differences in individual types of stressors between boys and girls (*p*=0.678).

## 4. Discussion

The primary finding of this study is that participants who reported AS in lessons did not have lower levels of PA during recesses or after school. Given the overall low PA of adolescents during recesses [38, 41], this finding highlights the major challenge of raising PA during recesses among all adolescents. Previous studies often note the presence of insufficient PA during recesses [42] and interventions aimed at increasing PA during recesses have been generally unsuccessful [43]. Most studies also indicate the failure of students to comply with the recommendation that PA should account for at least 50% of recess break [38]. In our study, PA during recesses represented 36.1% of recess time in boys and 31.0% in girls. This is consistent with our earlier research, in which we found that PA accounted for 46.3% and 35.2% of the overall recess time in boys and girls, respectively [38], and with similar studies conducted in secondary schools [44]. A differentiated approach to adolescents' PA during recesses according to their individual level of AS is unlikely to be successful in the school environment. Promotion of PA during recesses, after cognitively demanding lessons, may be more effectively implemented by involving school management and coordinating the educational program by teaching staff.

The crucial question is whether adolescents with recurrent AS should compensate for this mental load immediately after a lesson by a more intense but less time-demanding PA. Alternatively, such individuals could engage in more time-consuming forms of physically active recesses, as proposed by Pate et al. [45], i.e., at least once a day if a physical education lesson (PEL) is not scheduled. Our earlier finding that cumulative recess time exceeding 60 minutes cannot replace PELs regarding school PA is also important in this context [35]. However, the mental health benefits of short recesses remain very unclear compared to the health benefits arising from PELs.

There was statistically significantly lower PA among girls with AS than among boys reporting AS, as confirmed in selected PA characteristics during recesses but not in the after-school period. This does not contradict the recommendation to consider adolescent girls as an at-risk population that should be specifically supported by preventive mental health care [46]. Numerous studies indicate that older children and especially girls have a relatively high level of depression [47]. For instance, in 14/15-year-old girls, Poulsen et al. [48] observed significant associations between low level of leisure-time PA and poor MH at the age of 20/21 years.

That adolescents with AS are not more physically active after school than adolescents without AS represents a warning. There is a need to seek ways to promote PA among adolescents at mental health risk. Muller-Riemenschneider et al. [49] drew attention to the importance of improving conditions for healthy forms of recreation for rural adolescents to avoid boredom and involvement in activities that may not be safe or healthy. Similarly, Dagkas and Stathi [50] emphasized the need for the better and wider provision of structured physical activity in schools in economically deprived areas to compensate for lower participation levels in organized leisure-time PA. The environment is of critical importance for adolescents' PA in school, recreation, active transportation, and household [51]. Considering the gender specifications, the results of the present study indicate that it is desirable to focus on analyses of the associations of boys' AS and their participation in various sorts of organized PA. In girls, attention should be paid to the associations among AS, participation in organized PA, and tendencies of overweight or obesity.

PA is associated with decreased concurrent depressive symptoms in children [52], but it has not been established whether PA is protective against depressive symptoms in adolescents [53]. Stress and depression have an adverse effect on the level of PA and physical fitness in children [54]. Moreover, Fedewa and Ahn [55] discovered the positive effects of PA and physical fitness on children's achievement and cognitive outcomes. In particular, cardiorespiratory fitness can be an important indicator of health, including its mental dimension, in children and youth [30]. However, numerous studies have indicated the positive effects of PA in patients manifesting anxiety symptoms [56]. Thus, the associations of lifestyle-related variables and anxiety and stress-related variables with diastolic pressure and cardiovascular health must also be considered when investigating AS at lower-stage schools [57]. We also believe that adolescence and the time spent in school represent a “sensitive period” for the adoption of healthy work-related habits.

The individual responses of participants to recurring AS in four or more lessons were surprising. The most stress-responsive participants responded to AS with PA during recesses, although only at the level of the other study participants. From a physiological perspective, this was a rather insufficient response to mental load associated with high HR. Increased HR while physically inactive should be compensated for by increased HR during PA as suggested by Beijer et al. [58] who recommended high PA after prolonged television time with increased HR.

The PA of adolescents with AS should meet recommendations for specific segments of the day, i.e., recommendations regarding active transport to school, as well as PA at school, after school, and overall daily PA [38, 41]. Simplified recommendations of 60 min per day of PA every day [59, 60] or 11,000–14,000 steps per day [61, 62] are adequate for adolescents with AS. However, it is necessary to consider recommendations for moderate-to-vigorous PA, sedentary time, screen time, and sleep time [63].

As there are still no valid tools for measuring intrinsic, extraneous, and germane cognitive load [64], we chose the method of immediate self-assessment of MH immediately after the particular lesson, in order not to interfere with usual school conditions. During PA monitoring, cognitive stress assessment was a secondary task; therefore, an absolutely customary educational process took place for the participants in this study.

“Extraneous cognitive load is an element of interactivity that is caused by instructional factors and can be eliminated by altering instructional procedures” ([65]: 136). Associations between intrinsic and extraneous cognitive load are complex and therefore their clear distinction is not essential in educational practice. Most cognitive load effects, whether based on variations of intrinsic or extraneous cognitive load, may be explained using the common concept of elemental interactivity [65]. However, the identification of possible ways to eliminate undesirable stressors is essential in the educational process. According to the concept of van Merriënboer and Sweller [66], this concerns the transition from overload under the influence of extraneous load to decreased extraneous load and consequently to optimized germane load.

Repeated stressful situations, which are predominately initiated by test and examination procedures, call for change in evaluation techniques and optimization of their frequency within a single school day or week. It is equally important to promote PA-oriented integrated educational activities in seasonal, semiannual, annual, and multiyear cycles (thematically integrated activities, project days, sports courses, etc.). Addressing the impact of recurrent AS on sleep quality [67], negative moods [68], and other unwanted physiological responses, emotional reactions, and harmful behaviors [69] is a challenge for future interdisciplinary research on the biological, social, and environmental factors of adolescent development.

Evidence-based prevention programs that promote adolescents' MH and positive changes in the school environment should be implemented in schools [15, 70]. Programs for adults working with youth with MS-related issues are of comparable importance [71]. Based on the positive results of the Adolescent Depression Awareness Program intervention, Beaudry et al. [72] suggest such types of school preventive programs to improve depression literacy. Future studies of adolescents' MH in school should verify which programs increase “academic stress literacy” within physical and health literacy.

### 4.1. Strengths and Limitations

All-day objective monitoring of PA volume and intensity, using internal (HR) and external responses (METs) of the adolescent, in the context of subjective assessment of AS represents the main strength of the present study. Despite attempts to preserve customary school conditions, and without interfering with the school program, some disruption was inevitable due to wearing of chest straps, recording of time segments of the day, and self-assessment of AS. Although self-reported AS was confirmed by higher HR while being physically inactive, it is impossible to clearly characterize this mental load as distress. More in-depth analyses of the self-assessment of AS and physiological responses to stress or increased heart rate during physical inactivity were beyond the scope of the present study. A less positive attitude of boys than girls towards meeting the monitoring criteria and a smaller number of Polish than Czech research participants are further limitations of the study. This hinders options and scope for more detailed gender and country comparisons.

## 5. Conclusions

The study did not confirm the hypothesis that adolescents reporting AS would be less physically active during recesses or even after school, compared with adolescents without AS. However, the overall low PA of adolescents during recesses and after school highlights the need to compensate for AS by adequate PA, in particular among adolescents with recurrent AS in several consecutive lessons. School management and teaching staff should respect varying cognitive loads required by lesson content when preparing education programs. Programs should coordinate, restrict, or preferably use emotionally more acceptable forms of tests and exams, which are major causes of adolescents' AS. Recurring AS in consecutive lessons may lead to distress in adolescents, which may significantly contribute to worsening adolescents' MH. School management should ensure conditions that foster adolescents' PA during recesses, support the adoption of healthy habits to compensate for cognitive load during lessons, and promote AS literacy in adolescents. Along with parents, schools have the responsibility to promote in students the adoption of lifelong healthy working habits supportive of mental and physical health.

## Figures and Tables

**Table 1:** Sample characteristics.

Gender	*n*	Age (years)		Weight (kg)		Height (cm)		BMI (kg·m^−2^)		HRrest (beats·min^−1^)		Steps/day (number)	
		M	SD	M	SD	M	SD	M	SD	M	SD	M	SD
Boys	187	16.43	1.17	70.33	12.42	178.40	7.90	22.04	3.30	60.53	6.04	9373	4172
Girls	339	16.39	1.17	59.39	9.54	167.71	6.78	21.11	3.14	63.08	6.66	8955	3370

M: mean; SD: standard deviation; BMI: body mass index; HRrest: resting heart rate.

**Table 2:** Overall physical activity during recesses of adolescents with and without academic stress.

PA characteristics	Boys				Girls				*H*	*p*	*η* ^2^
	Without AS (*n* = 244)		With AS (*n* = 95)		Without AS (*n* = 547)		With AS (*n* = 236)				
	Mdn	IQR	Mdn	IQR	Mdn	IQR	Mdn	IQR			
Physical activity (min·hour^−1^)	34.57	15.98	37.01	14.24	31.06	11.47	30.60	11.40	34.54^a^	<0.001	0.066^*∗∗*^
Steps·hour^−1^ (number)	1081	581	1160	861	961	554	936	470	18.23	<0.001	0.035^*∗*^
MVPA ≥ 3 METs (min·hour^−1^)	4.20	4.95	5.08	5.96	3.00	4.06	3.30	3.83	26.55	<0.001	0.051^*∗*^
MVPA ≥ 60% HRmax (min)	0.50	4.43	1.50	7.39	1.12	5.31	1.64	5.40	2.30	0.513	0.004

AS: academic stress; MVPA: moderate-to-vigorous physical activity; Mdn: median values; IQR: interquartile range; *H*: Kruskal-Wallis test; *η*^2^: Cohen´s effect size; *p*: significance level; *η*^2^: ^*∗*^0.01 ≤ *η*^*2*^ < 0.06 small effect size; ^*∗∗*^0.06 ≤ *η*^2^ < 0.14 medium effect size; a: significant difference between groups (boys versus girls with AS).

**Table 3:** Overall after-school physical activity in adolescents with and without academic stress.

PA characteristics	Boys				Girls				*H*	*p*	*η* ^2^
	Without AS (*n* = 244)		With AS (*n* = 95)		Without AS (*n* = 547)		With AS (*n* = 236)				
	Mdn	IQR	Mdn	IQR	Mdn	IQR	Mdn	IQR			
Physical activity (min·hour^−1^)	22.91	14.96	22.12	13.28	24.22	9.98	24.28	11.15	3.63	0.304	0.007
Steps·hour^−1^ (number)	601	603	716	613	670	549	814	530	14.76^d^	0.002	0.028^*∗*^
MVPA ≥ 3 METs (min·hour^−1^)	3.38	4.33	4.22	4.29	3.00	3.90	4.35	4.23	15.36^d^	0.002	0.029^*∗*^
MVPA ≥ 60% HRmax (min)	0.47	2.96	0.53	1.46	0.59	1.53	0.65	1.98	3.23	0.357	0.006

AS: academic stress; MVPA: moderate-to-vigorous physical activity; Mdn: median values; IQR: interquartile ranges; *H*: Kruskal-Wallis test; *η*^2^: Cohen's effect size; *p*: significance level; *η*^2^: ^*∗*^0.01 ≤ *η*^2^ < 0.06 small effect size; ^*∗∗*^0.06 ≤ *η*^2^ < 0.14 medium effect size; d: significant difference between groups (boys versus girls with AS).

**Table 4:** Meeting the 6000 steps after school time recommendation in boys and girls with or without academic stress (AS) in school.

Variables	Boys				Girls			
	Model 1		Model 2		Model 1		Model 2	
	OR (95% CI)	*p*	OR (95% CI)	*p*	OR (95% CI)	*p*	OR (95% CI)	*p*
*Academic stress*								
With AS ref. Without AS								
	0.795 (0.395–1.599)	0.519	0.609 (0.274–1.352)	0.223	2.934 (1.673–5.146)	<0.001	3.183 (1.734–5.815)	<0.001

*Age (years)*								
<16 ref.								
16			1.283 (0.551–2.987)	0.563			1.132 (0.641–1.999)	0.669
>16			0.844 (0.318–2.245)	0.735			0.952 (0.497–1.8225)	0.882

*BMI*								
≥25 kg m^−2^ ref.								
<25 kg m^−2^			0.729 (0.275–1.930)	0.525			0.454 (0.206–1.001)	0.050

*Participation in organized PA*								
No ref.								
Yes			2.616 (1.127–6.074)	0.025			0.624 (0.367–1.061)	0.081

*Day of the week*								
Friday ref.								
Thursday			0.710 (0.259–1.945)	0.506			1.204 (0.608–2.384)	0.533
Wednesday			0.695 (0.196–2.459)	0.572			1.00 (0.462–2.168)	0.999
Tuesday			0.563 (0.180–1.756)	0.322			0.744 (0.328–1.689)	0.480
Monday			0.323 (0.089–1.728)	0.217			0.612 (0.236–1.587)	0.313

*Country*								
Czech rep. Ref.								
Poland			0.685 (0.227–2.063)	0.501			0.647 (0.290–1.444)	0.288

*Residence (population)*								
<1,000 ref.								
1,000–29,999			0.658 (0.265–1.636)	0.368			0.893 (0.495–1.612)	0.708
30,000–100,000			1.659 (0.560–4.915)	0.361			0.606 (0.293–1.255)	0.178
>100,000			0.836 (0.194–3.605)	0.810			0.452 (0.143–1.422)	0.174

*Dog ownership*								
No ref.								
Yes			1.115 (0.552–2.251)	0.765			0.919 (0.556–1.518)	0.742

*Note*. OR = odds ratio; CI = confidence interval; statistical significance of *p* < 0.05; Model 1 = academic stress; Model 2 = adjusted for age, BMI, participation in organized PA, day of the week, country, size of the place of residence, and dog ownership.

## Data Availability

The data used to support the findings of this study are available from the corresponding author upon request.
